# Human infection with pseudorabies virus: review of evidence, risk assessment, and future directions

**DOI:** 10.1128/jvi.00080-26

**Published:** 2026-04-29

**Authors:** Herman W. Favoreel

**Affiliations:** 1 Department of Translational Physiology, Infectiology and Public Health, Faculty of Veterinary Medicine, Ghent University 26656, Ghent, Belgium; Indiana University Bloomington, Bloomington, Indiana, USA

## Abstract

Pseudorabies virus (PRV) is an alphaherpesvirus with a broad host range, with the pig as its natural host. Although PRV can use human cell surface receptors for cell entry, humans and higher primates have been considered as refractory or showing very limited susceptibility to PRV infection. Between 1914 and 2011, only 17 cases of suspected human PRV infection were described, typically displaying temporal and mild/intermediate disease symptoms, mostly consisting of severe itching. No neurological complications were reported. In addition, in 1970, one researcher conducted a repetitive PRV self-inoculation experiment, which resulted in neither symptoms nor seroconversion. However, since 2011 and mostly based on next-generation sequencing on cerebrospinal fluid, 34 human cases of suspected PRV infection have been reported, all of which were linked with severe, life-threatening encephalitis and/or visual impairment. All these cases were reported in China, where there has been a concomitant surge in outbreaks of PRV strains that show genetic differences compared to classical PRV strains. All suspected recent cases of human PRV infection had (occupational) contact with raw pig meat and often had skin damage on the hands or fingers, or direct eye contact with potentially PRV-infected material. Importantly, human-to-human PRV infection has never been reported. Although the risk for human PRV infection remains very low, these recent developments should guide increased efforts to eradicate PRV from domestic pig populations, increase protection of individuals at risk, and increase research on improved diagnostics, effective antivirals, and a better understanding of the host and viral factors that determine human susceptibility or resistance to PRV infection.

## PSEUDORABIES VIRUS (PRV)

Pseudorabies virus (PRV), also called Aujeszky’s disease virus or suid alphaherpesvirus 1, belongs to the alphaherpesvirus subfamily of the herpesviruses, which includes several other members such as human herpes simplex virus (HSV) 1 and 2 and varicella-zoster virus (VZV).

Members of the *Sus scrofa* species represent the natural reservoir of PRV, and these include domestic pigs, wild boar, and feral swine. PRV-associated disease in pigs is remarkably age-dependent. Infected suckling piglets display severe nervous system disorders and usually die within 1 or 2 days, with mortality rates reaching 100%. Weaned piglets, aged 3 to 9 weeks, also display severe neurological disease but substantially lower mortality rates. Older pigs typically do not develop overt neurological symptoms upon PRV infection but often present with respiratory illness and reproductive disorders. Although disease symptoms are less prominent in PRV-infected older pigs, infection often leads to reduced body weight and consequently substantial economic loss for swine producers (1, 2). Following acute infection, PRV establishes lifelong latency in the surviving host, particularly in sensory peripheral neurons of the trigeminal ganglion. During latency, the viral genome persists as an intranuclear episome without integration in the host genome, and transcriptional activity is limited to the expression of so-called latency-associated transcripts. Stressful experiences (e.g., vehicular transport, concomitant disease) or treatment with immunosuppressive agents may trigger reactivation from latency and recurrent virus replication, excretion, and transmission (1).

Alphaherpesviruses in general have a broader host tropism compared to beta- or gammaherpesviruses, although the host range of PRV is particularly large (1). Indeed, PRV has been reported to infect a wide range of domestic and wild mammals, including cattle, sheep, dogs, cats, rabbits, rodents, and raccoons, and even very young chickens show some susceptibility (1, 3–5). In fact, PRV-induced disease (“mad itch”) was first described in cattle in the 19th century, long before PRV was recognized as a swine pathogen (1, 2, 6). In non-natural susceptible hosts, PRV infection typically results in rapid and very severe neuropathology, in particular insatiable itching, and is almost invariably lethal (5). Historically, humans and non-human primates have been largely considered refractory or displaying very low susceptibility to PRV infection (1).

Due to the severe health and economic impact of PRV on the swine industry, several countries and regions, particularly in Europe and the USA, have initiated stringent and mandatory mass vaccination and eradication programs (6, 7). Eradication programs were first initiated in the 1970s and 1980s in Europe and the USA, reaching their full impact in the 1990s and early 2000s, which led several countries, particularly in western Europe, to achieve an official PRV-free status (6, 7). An important point to make is that a PRV-free status reflects the lack of PRV in the domestic pig population but does not necessarily mean that a country is free of circulating PRV strains in the wild boar population. Infected wild boar populations, therefore, represent a possible reservoir for the reintroduction of PRV in the domestic pig population (8). Of note, there are reports indicating that PRV strains circulating in wild boar display reduced virulence in domestic pigs compared to strains circulating in the domestic pig population (9–11).

A critical part of the success of the PRV eradication programs has been the development and introduction of the so-called marker vaccine and DIVA concept, which stands for “differentiate infected from vaccinated animals.” Attenuated PRV vaccine strains that were developed lacked important virulence genes, either through classical *in vitro* virus passaging or via specific genetic engineering. Since some of these virulence genes encode immunogenic proteins, in particular the viral gE glycoprotein, these vaccines not only are attenuated and generate a protective immune response but also allow for discriminating infected from vaccinated animals based on serological differences. Indeed, diagnostic ELISA tests allow differentiation of PRV-infected (PRV antibody positive and gE antibody positive) animals from vaccinated but PRV-uninfected (PRV antibody positive but gE antibody negative) animals. The combination of efficacious vaccines and accurate differential ELISAs to identify infected versus vaccinated animals has been crucial in the development of successful PRV eradication programs (6, 7).

Not all PRV-affected countries with substantial pig production have initiated stringent eradication programs, and as a consequence, PRV currently still causes substantial outbreaks and associated economic losses in different countries and regions worldwide, most notably in China (12). Of note, PRV strains that currently circulate in China display genetic differences compared to classic PRV strains that have been isolated in other countries, particularly in Europe and the USA. Based on genome sequence differences, including in genes encoding viral glycoproteins, PRV strains can be subdivided into two groups, designated as genotype I and genotype II (13). Genotype II currently only harbors PRV strains that have been isolated in China. Although the amino acid differences in PRV glycoproteins in genotype I versus II strains are limited, there are indications that some of these may result in antigenic differences (14). Some data also suggest that genotype II strains can be genetically further subdivided into a 2.1 clade and 2.2 clade, with older Chinese genotype II strains belonging to the 2.1 clade, whereas more recent Chinese PRV strains isolated since 2011 typically belong to the 2.2 clade and are sometimes referred to as “variant PRV strains” (13). Since 2011, there have been increasing reports of large PRV outbreaks in pig farms in China associated with these variant PRV strains, some of which have been claimed to break immunity provided by (genotype I-based) PRV vaccines, including the most commonly used attenuated PRV vaccine strain, Bartha (13). Some variant PRV strains in China also appear to display increased virulence towards pigs in experimental settings, causing substantial neurovirulence and even mortality in older pigs (15, 16).

## HUMAN INFECTION WITH PRV: THE THEORY

Whether a species is susceptible to a specific virus infection in many cases depends to a large extent on the presence or absence of cell surface receptors that the virus engages to gain entry in host cells. For example, the ability of the S envelope protein of SARS-CoV-2 to interact with different species’ orthologs of the ACE2 (angiotensin-converting enzyme 2) cell surface receptor correlates well with the susceptibility of these specific species to SARS-CoV-2 infection (17–19).

Cell entry of PRV, like that of the related human HSV1 and HSV2 viruses, requires binding of the viral gD envelope glycoprotein to one of its entry receptors, including nectin-1, nectin-2, or poliovirus receptor (PVR) (20, 21). PRV gD can interact with human orthologs of these receptors and use them to gain entry into host cells (20, 21). At least for nectin-1, which is thought to be the most effective gD receptor, the affinity of PRV gD for this protein is similar for the human and swine orthologs (22). Hence, the lack of a functional gD receptor does not explain humans’ low susceptibility to PRV infection. In line with this, different human immortalized cell lines, including widely used HeLa and HEK293 cells, are readily susceptible to PRV infection *in vitro*. Nonetheless, older literature indicates that, although primary human embryonic fibroblasts are also susceptible to PRV infection, primary human lung fibroblasts display reduced susceptibility to PRV infection, and other primary human cells, including human skin muscle cells, human stromal cells, and human kidney cells, do not appear to be susceptible to PRV infection (23).

The somewhat inconvenient truth is that we currently do not really understand why humans and higher primates display high resistance to PRV infection. However, in addition to the putative low susceptibility of different primary human cells to PRV infection, some hypotheses can be put forward.

One important aspect of general human protection against enveloped viruses that originate from non-primate mammals comes from the fact that humans and primates contain naturally occurring antibodies directed against specific carbohydrate epitopes, in particular Galα1-3Galβ1-4GlcNAc-R (α-Gal) and, to a lesser extent, the sialic acid N-glycolylneuraminic acid (Neu5Gc) (24). This is due to evolutionary inactivation in humans and primates of the enzymes that generate these glycan moieties, α1,3-galactosyltransferase (GGTA1) and cytidine monophosphate-N-acetylneuraminic acid hydroxylase (CMAH), respectively (24). In healthy humans, anti-α-Gal antibodies are produced continuously, largely in response to α-Gal-bearing commensal gut bacteria. They constitute up to 1–5% of circulating antibodies (IgG and IgM combined) and thus represent one of the most abundant single antigen-specific antibody populations in humans (25). Anti-Neu5Gc antibody levels are lower and more variable and are typically generated by dietary exposure (26). In species that do express these glycans, enveloped viruses typically carry these moieties on viral envelope glycoproteins, leading to their recognition and fast neutralization and/or elimination by this human natural immune response (24). One important consequence hereof is that growing viruses that originate from non-primate mammals, such as PRV, in human cell cultures *in vitro* may lead to the generation of progeny virus particles that are devoid of these human immune system-activating glycans and, therefore, may pose an increased risk of human infection. Hence, it is advisable to impose proper biosafety protocols when doing infection assays with PRV or other non-primate viruses in human or primate cell cultures.

Although speculative, an additional factor that may contribute to humans’ increased resistance to PRV infection could be some cross-protective immunity provided by human alphaherpesvirus infection. In line with this, cross-reactive antibodies against alphaherpesviruses from different species, and even cross-neutralizing antibodies against HSV and PRV, have been reported before (27, 28). Arguing against this possibility, other species that serve as hosts for closely related alphaherpesviruses, such as cattle and dogs, are readily susceptible to PRV infection. However, the prevalence of bovine alphaherpesvirus 1 (BoHV-1) and canine alphaherpesvirus 1 (CHV-1) is much more variable and substantially lower on a global scale than that of human alphaherpesviruses (29, 30). Indeed, seroprevalence of human alphaherpesviruses is uniformly high across the world population with estimates of 67%, 13%, and 90% for HSV1, HSV2, and VZV, respectively (31, 32).

## SUSPECTED HUMAN PRV INFECTIONS BEFORE 2011

Before 2011, 17 suspected cases of human PRV infection were reported in scientific literature (33–37), all associated with temporal and relatively mild to intermediate disease symptoms. This time period also contains a report of an infamous voluntary PRV self-inoculation experiment that did not result in disease symptoms or detectable seroconversion (38) (Table 1).

**TABLE 1:** Suspected cases of human PRV infection before 2011 (ordered chronologically)^*a*^

Case	Year	Sex	Occupation	Serology (neutralizing antibodies)	Symptoms	Symptom duration	Reference
1	1914	?	Lab tech	ND	Severe itching, sore throat, weakness, restlessness	Days	(33)
2	1914	?	Lab tech	ND	Severe itching, sore throat, weakness, restlessness	Days	(33)
3	1940	M	Lab tech	ND	Pruritus, skin erythema, weakness	1–2 days	(34)
4	1940	F	Lab tech	ND	Severe itching, headache, dizziness	3 days	(34)
5	1963	M	Animal caretaker	ND	Severe sore throat, leg weakness, insomnia	Days	(35)
6	1963	M	Animal caretaker	ND	Severe sore throat, leg weakness, insomnia	Days	(35)
7	1963	M	Night watchman	ND	Severe sore throat, leg weakness, insomnia	Days	(35)
8	1963	M	Veterinarian	ND	Severe sore throat, leg weakness, insomnia	Days	(35)
9	1970	M	Scientist	Negative	Repeated self-inoculations: no symptoms	–	(38)
10	1983	M	? contact with ill cat	Neutralizing antibodies	Local inflammation, muscle/joint pain, headache, hypersalivation, weight loss	±1 year	(36)
11	1985	M	? contact with cats	Neutralizing antibodies	Fever, weakness, dysphagia, altered smell/taste	Months	(36)
12	1985	F	? contact with cats	Neutralizing antibodies	Fever, weakness, dysphagia, altered smell/taste	Months	(36)
13	1992	M	Cattle farm	Negative	Severe itching, periodic weakness, heavy sweating	Months	(37)
14	1992	M	Cattle farm	Negative	Severe itching	Days, periodic recurrence	(37)
15	1992	M	Cattle farm	Negative	Severe itching	Days	(37)
16	1992	M	Cattle farm	Negative	Severe itching	Days	(37)
17	1992	M	Cattle farm	Negative	Severe itching	Days	(37)
18	1992	M	Cattle farm	Negative	Severe itching	Days	(37)

^*a*^ –, no symptoms; ?, unknown; ND, not determined.

In 1914, only 12 years after Aladár Aujeszky first isolated the infectious agent that caused pseudorabies, which was eventually named after him (39), the first report appeared of two cases of suspected human PRV infection (33). In both cases, lab technicians that had wounds on their hands were directly exposed to infectious material: one necropsy assistant injured himself while skinning an animal, while another employee accidentally punctured his skin with a syringe filled with infectious brain suspension. In both cases, the lab technicians experienced severe itching, weakness, restlessness, and a sore throat for about 60–70 h.

A second report was published at the beginning of the Second World War and again involved two lab technicians who injured one of their hands while working with PRV-infected material. In both cases, the contaminated material was derived from PRV-infected dogs (34). In one case, symptoms consisted of pruritus and skin erythema for 24 h and general weakness the following day, after which the symptoms resolved. In the other case, the affected person experienced itching on the injured hand and arm, which spread to the shoulder with progressively increasing intensity over 48 h. Afterward, the patient experienced a headache and dizziness, skin erythema on the legs, and aphthous lesions on the oral mucosa but was completely cured within 3 days. Of interest, blood that was drawn from the latter person at the time of intense itching was injected in rabbits, which died within 18 h with disease symptoms reminiscent of those of a PRV infection. As such, these data for the first time point to the possibility of infectious PRV in the blood of a human suspected of being infected with PRV. When blood was again drawn from the same patient at 8 days after the presumed infection and was subsequently injected in rabbits, the animals did not develop disease symptoms (34).

In 1963, Hussel et al. described four suspected cases of human PRV infection (35), where human disease symptoms occurred a few days after a PRV outbreak on a pig farm. Two animal caretakers, a night watchman (whose dog had died because of PRV infection), and an attending veterinarian all suffered from a severe sore throat, insomnia, and leg weakness for a couple of days.

In an attempt to try to resolve the controversial issue of whether humans are susceptible to PRV infection, Jentzsch and Apostoloff (1970) tested 445 human sera for the presence of PRV-neutralizing antibodies (38). Sera were mostly obtained from people with occupations involving raw pig meat handling, including 223 blood samples from slaughterhouse and meat-processing personnel. None of the sera showed any neutralizing activity against PRV, while positive control samples from PRV-infected pigs did. Of anecdotal interest, one of the (negative) sera was from Dr. C.H. Becker, a veterinarian who, without specific protective equipment, investigated several aspects of PRV pathogenesis and *in vitro* replication characteristics and who isolated the PRV strain Becker, which is still widely used in laboratories all over the world (40).

In a quite dramatic turn of events, based on these negative human serum test results, one of the authors of the Jentzsch and Apostoloff (1970) study decided to perform a self-inoculation experiment, consisting of an intradermal injection with 10^3.4^ TCID_50_ virulent PRV at the inner surface of the forearm (38). Apart from slight redness at the injection site, no objective or subjective symptoms were observed, and no PRV-neutralizing antibodies were detected in blood samples taken at 21 and 37 days post-inoculation (dpi), while a rabbit inoculated intramuscularly with the same infectious dose died within 3 days. To validate these findings, a second self-inoculation was performed, consisting of repeated subcutaneous injection of 0.1 mL, 0.2 mL, and 0.5 mL of virus (at a concentration of 10^5.5^ TCID_50_ PRV/0.2 mL) with intervals of 3 and 7 days. Again, apart from slight redness at the injection sites, no reactions occurred, and no neutralizing antibodies could be detected in blood samples taken at 8, 18, and 31 dpi. Subcutaneous injection of 0.2 mL of the same culture fluid killed a rabbit in a little over 2 days (38). As far as known, this is the only deliberate experimental human PRV inoculation ever reported. In addition to providing some of the strongest support for the low susceptibility of humans to PRV infection, the assay certainly demonstrated that the 1960s and 1970s were wild.

In 1987, Mravak et al. reported in *The Lancet* three suspected cases of human PRV infection, who were thought to have contracted the disease from cats (36). One case consisted of a 52-year-old male who had contact with a severely ill cat and who cut his left thumb while washing the cat’s dishes. After a week, the thumb became inflamed, and the person felt weak and feverish. The following weeks, he suffered from muscle and joint pain and headaches, hypersalivation, weight loss, loss of tactile sense, and a feeling of inner tension. The intensity of the symptoms decreased slowly, with several relapses over a period of almost a year. The other two cases concerned a 43-year-old male and his 41-year-old wife, who had close contact with cats that may or may not have been diseased. Both have fallen ill with, among other symptoms, tiredness, fever, dysphagia, and the perception of strange smells and tastes. After several months, both had recovered completely. Importantly, serum samples of all three cases were tested (between 5 and 15 months after onset of clinical illness) and were (weakly) positive for neutralizing antibodies against PRV. Two to 24 months later, patients were seronegative again (36). This represents the first report of (low levels of) neutralizing antibodies against PRV in the serum of human patients suspected of PRV infection and, therefore, represents the first direct experimental evidence that humans may display some susceptibility to PRV infection.

In 1992, Anusz et al. reported six suspected human cases of PRV infection during a large PRV outbreak in a beef bull farm in Poland (37). A couple of days after the disease onset in the animals, clinical symptoms suggestive of PRV appeared in workers that had direct contact with the affected cattle. Family members and other farm workers remained unaffected. The symptoms mostly consisted of severe itching of the hands, sometimes spreading to the arms, shoulders, and the head that lasted for several days. Two of the affected workers experienced periodic recurrences of the itching. One of the affected workers, who had the longest contact with the diseased cattle and spent almost the entire duration of the PRV outbreak in the barn, also suffered from periodic weakness and heavy sweating, as well as recurrent skin rashes and itching around the eyes and scalp that lasted for months. However, serological tests on 22 employees of the farm, including the affected farm workers, were negative for PRV-specific antibodies. A more extensive serological survey of 62 employees on other farms revealed five positive reactions, four of which had a very low titer (37).

Summarizing this period, 17 reported cases of suspected human PRV infection in a period spanning almost 100 years (1914–2011) is a strong indicator for the high resistance of humans against PRV infection and disease. This is especially true considering that this time period encompasses the large outbreaks and worldwide exponential spread of PRV, which were associated with the intensification of pig breeding from the 1960s onward (1, 2). This exponential spread of PRV undoubtedly has led to very frequent contact of many people (including farmers, animal caretakers, veterinarians, slaughterhouse personnel, butchers, and scientists) with high titers of infectious virus but was not associated with an obvious increase in suspected human PRV infection cases.

In addition, for most of these few cases in the period 1914–2011, direct evidence of PRV infection is lacking, and suspicions are mostly based on symptomatic humans coinciding with (suspected) PRV outbreaks in livestock or other animals. Only three cases of suspected field infections were associated with (low levels of) PRV-neutralizing antibodies in serum during this period (36). Other suspected cases from this period were either negative or not tested for PRV-specific antibodies (Table 1). Furthermore, a self-inoculation experiment did not result in either disease symptoms or detectable seroconversion (38).

Nonetheless, assuming that all these 17 cases indeed would represent rare but *bona fide* human infections, PRV infection during this period generally resulted in relatively mild to intermediate disease symptoms, most often including severe itching, which typically resolved within days, although in some cases it may have lasted for months to a year. No encephalitis-like symptoms, long-term neurological sequelae, or visual impairment were reported in any of these cases. Of importance, as far as we know, none of these suspected cases of human PRV infection were treated with antiviral drugs such as acyclovir, foscarnet, or ribavirin. The antiviral activity of these drugs against HSV was only discovered in the 1970s (41–43). Only several years later, they were found to also display antiviral activity against PRV (44–47). However, most assays testing their antiviral activity against PRV were performed *in vitro,* and the effectivity of at least some of these drugs against PRV appears to be substantially lower compared to their activity against HSV (44, 48, 49).

## SUSPECTED HUMAN PRV INFECTIONS SINCE 2011

Since 2011, 34 suspected cases of human PRV infection were reported, and all of these were associated with severe encephalitis and/or vision loss (50–71). All 34 cases were reported in China, with the oldest (retrospectively analyzed) case dating back to 2011 (57), which coincides with the increasing reports of PRV outbreaks and the identification of different PRV variants in China (72). In all cases, suspicion of PRV infection was based on next-generation sequencing (NGS) analysis, mostly of cerebrospinal fluid (CSF) but also of vitreous humor of the eye and/or blood (Table 2). There is a large variety in NGS results, with the detection of unique PRV reads ranging from only 1 to over 23,000 reads, spanning 0.02 to 86.0 % of the PRV genome (Table 2). As indicated further, all patients were treated with antivirals, which may possibly have affected the number of virus sequence reads detected by NGS. In some cases, NGS results were confirmed by PCR and/or Sanger sequencing (50, 53, 56, 57, 61, 62). In addition, PRV-specific antibodies were detected in serum and/or CSF in 14 cases (Table 2). PRV-neutralizing antibodies were detected in two reports, while eight reports mention ELISA-based detection of gB-specific antibodies and five mention gE-specific antibodies. Importantly, in one case, a PRV strain designated hSD-1/2019 was isolated from the CSF of the patient (60).

**TABLE 2:** Suspected cases of human PRV infection since 2011 based on detection of PRV sequences via NGS (ordered chronologically based on publishing date)^*c*^

Case	Year estimate based on publication year^*a*^	Age	Sex	Occupation	NGS CSF/eye/blood PRV reads (% PRV genome coverage)	Serology serum/CSF gB/gE ELISA neutralizing antibodies	Enceph.	Visual disability/vision loss	Long-term outcome	Reference
1	2017	45	F	Swineherd	Eye 4,832 (84%)	Serum, CSF gB S/*N* < 0.6	–	+ endopht.	Partial recovery	(50)
2	2016–2017^*a*^	55	M	Pork dealer	CSF 20 (1.6%)	Serum, CSF gB S/*N* < 0.6 gE S/*N* < 0.6	+	–	Deceased	(51, 57)
3	2016–2017^*a*^	51	M	Cook	CSF 242 (16.0%)	Serum, CSF gB S/*N* < 0.6 gE S/*N* < 0.6	+	–	Deceased	(51, 57)
4	2016–2017^*a*^	38	M	Butcher	CSF 6 (0.2%)	Serum gB S/*N* < 0.6	+	–	Severely incapacitated	(51, 57)
5	2018^*a*^	59	M	Swineherd	CSF 8 (?%)	Serum neutr. abs	+	+	Partial recovery	(52)
6^*b*^	2018^*a*^	43	M	Veterinarian	CSF 6,198 (80.6%)	Serum gB S/*N* < 0.3	+	–	Coma	(53, 60)^*b*^
7	2018	50	M	Pig slaughterer	CSF 37 (3.9%)	ND	+	–	Moderate disability	(54)
8	2018	50	F	Pork cutter	CSF 20 (1.9%)	ND	+	–	Ventilator support	(54)
9	2018	43	M	Sick pig handler	CSF 72 (3.4%)	ND	+	+	Partial recovery	(54)
10	2018	59	M	Pork cutter	CSF 16 (0.4%)	ND	+	–	Ventilator support	(54)
11	2018	50	M	Pork cutter	CSF 2 (0.1%)	ND	+	+	Partial recovery	(54)
12	2018–2019^*a*^	44	M	Pig handler	CSF 72 (3.4%) eye 2 (0.03%)	ND	+	+ ARN	Partial recovery	(55)
13	2019	44	M	Pork vendor	CSF 74 (2.5%) blood 3 (0.1%)	Serum, CSF	+	–	Partial recovery	(56)
14	2018	Mid age	M	Butcher	CSF 141 (10.9%)	Serum gB S/*N* < 0.6	+	–	Deceased	(57)
15	2018	Mid age	M	Swineherd	CSF 30 (2.5%)	CSF gE S/*N* < 0.6	+	+	Partial recovery	(57)
16	2018	Young	M	Driver	CSF 52 (1.9%)	Serum gB S/*N* < 0.6 gE S/*N* < 0.6 CSF gB S/*N* < 0.6	+	–	Partial recovery	(57)
17	2011	Young	F	Pork dealer	CSF 64 (4.9%)	Negative (serum and CSF)	+	–	Deceased	(57)
18	2019–2020^*a*^	49	M	Pig slaughterer	CSF negative eye 153 (4.3%)	ND	+	+ ARN	?	(58)
19	2020	40s	M	Livestock breeder	CSF 68 (?%) eye 23,277 (?%)	ND	+	+ endopht.	Partial recovery	(59)
20	2018	25	M	Veterinarian	CSF 8 (0.6%)	Serum gB S/*N* < 0.4 gE S/*N* < 0.6 neutr. abs	+	+	Partial recovery	(60)
21	2019	35	M	Butcher	CSF 7 (0.2%)	ND	+	–	Partial recovery	(60)
22	2019	49	M	Butcher	CSF 43 (1.5%)	ND	+	–	Minimally conscious	(60)
23	2021	54	M	Butcher	CSF 652 (25.3%) eye 1,566 (35%) blood 1 (-)	ND	+	+ endopht.	Deceased	(61)
24	?	51	F	Housewife (raw pig meat contact)	CSF 34 (2.4%)	ND	+	–	Severely incapacitated	(62)
25	?	68	M	Swineherd	CSF 29 (2.6%)	ND	+	–	Deceased	(62)
26	2021–2022^*a*^	42	M	Pork seller	CSF 13,996 (86.0%)	ND	+	+	Partial recovery	(63)
27	2019	43	M	Pig farmer	CSF 1 (0.03%)	ND	+	+	Partial recovery	(64)
28	2021	54	M	Pig industry	CSF 41 (1.9%)	ND	+	+	Partial recovery	(65)
29	?	44	M	Pig seller	CSF 83 (?%)	CSF	+	–	Partial recovery	(66)
30	2021–2022^*a*^	51	F	Pork dealer	CSF 349 (?%)	ND	+	–	?	(67)
31	2022–2023^*a*^	56	M	Butcher	CSF negative eye 4 (0.02%)	serum gB S/*N* < 0.4	+	+	Partial recovery	(68)
32	2023–2024^*a*^	52	F	?	CSF ? (?%) eye ? (?%)	ND	+	+ ARN	?	(69)
33	2021	40	M	Chef	CSF 657 (?%) eye 3,609 (?%)	ND	+	+ endopht.	Complete recovery	(70)
34	2019	35	M	Pig industry	CSF ? (?%)	ND	+	+	Partial recovery	(71)

^*a*^ In these cases, the year was estimated based on the publication as no specific date was mentioned in the publication.

^*b*^ Case number 6: based on the description of this case (including hospital, details about the patient, number of unique PRV reads, percentage of genome coverage), we assume that case 4 in reference 60 is the same case as the case that was described earlier in reference 53.

^*c*^ ARN, acute retinal necrosis; endopht., endophthalmitis. –, symptom not reported; +, symptom reported; ?, unknown; ND, not determined.

In all the reported human cases of suspected PRV infection since 2011, the disease onset involved flu-like symptoms for one or several days, particularly (intermittent) fever and headache. This was typically followed by loss of responsiveness and seizure episodes consisting of face switches, involuntary limb jerks, upward turning of the eyes, and/or incontinence. Subsequently, the condition of most patients rapidly deteriorated, with respiratory failure requiring mechanical ventilation and, in several cases, leading to coma. All cases except one were diagnosed with encephalitis, typically with very severe and long-term neurological sequelae, ranging from moderate to severe mental and physical disability (most cases) to permanent coma or unresponsiveness (*n* = 4), and sometimes death (*n* = 6) (Table 2). Only one case described complete recovery of the patient (70).

Clinical presentation and characteristics of the CSF show similarity with Japanese encephalitis (JE) or herpes simplex encephalitis (HSE) (53). Brain MRI imaging may help to discriminate between these different conditions. MRI data of encephalitis cases suspected to be associated with PRV infection typically reveal panencephalitis, simultaneously involving the limbic system, basal ganglia, and brainstem. This is unlike HSE, where lesions are primarily confined to the limbic system and do not extend to the basal ganglia and, to some extent, also unlike JE, which predominantly involves symmetric thalami and substantia nigra (51, 53). Secondary cerebral hemorrhage appears to be less prominent in PRV-associated cases versus HSE (51, 53).

From a certain point in their hospital trajectory onward, all patients were treated with antivirals, most often consisting of systemic treatment with acyclovir (or sometimes ganciclovir) as a monotherapy or in combination with foscarnet or ribavirin. In addition, medications to suppress seizures (typically midazolam and sodium valproate), reduce inflammation (corticosteroids), lower intracranial pressure (mannitol), and/or support the immune system (human immunoglobulins) were also administered. The effects of antiviral treatment on the course of encephalitis and general disease status of the patients were largely disappointing, although particularly a ganciclovir and (sometimes intravitreal) foscarnet combination therapy in some cases was associated with some improvement of the clinical condition of the patient and a gradual reduction of PRV NGS reads in the CSF and/or aqueous humor to undetectable levels (63, 70, 71).

Several cases were associated with severe eye damage, including endophthalmitis and/or acute retinal necrosis (ARN), leading to severely and mostly permanently impaired vision or blindness (Table 2). This was reported for 17 out of 34 patients, although this may be an underestimation, as patients who remained comatose or died were often not carefully assessed for possible eye damage. Of note, in several cases, the appearance and/or aggravation of ocular symptoms was reported only one to four months after disease onset, despite prolonged antiviral treatment (54, 55, 57, 58, 64, 65, 68). Despite the hallmark “mad itch” caused by PRV in susceptible non-natural host species, and the itching observed in several suspected cases of human PRV infection before 2011 (Table 1), itching was not described in the suspected human PRV infection cases since 2011.

All reported patients had occupational or other frequent contact with raw (and possibly PRV-infected) pig material (Table 2). Several case studies (11/34) explicitly mention that the patient had skin damage on the hands and/or fingers around the time of supposed contact with PRV-infected material (51–54, 56, 61, 68, 71). One somewhat different case was associated with endophthalmitis without indication of encephalitis, which was suggested to be due to direct eye contact with (possibly PRV-contaminated) sewage from a hog farm (50). Although there were no indications for CNS involvement in this particular patient, four of the patient’s CSF samples were considered positive for gB-specific antibodies (gB S/*N* < 0.6) (50).

## CONCLUDING REMARKS AND KNOWLEDGE GAPS

Although many questions remain, based on the available evidence, it is reasonable to assume that, in very rare cases, PRV may infect humans. Cases reported since 2011 were all based on NGS detection of PRV reads in the CSF, eye, and/or blood of patients, and all patients were reported in China. Importantly, the outcome of these more recent suspected human PRV infections is much more devastating and life-threatening compared to the temporal and relatively mild or intermediate symptoms associated with the very few older cases of suspected human PRV infection. Importantly, human-to-human spread of PRV has never been reported.

Although several of the recent suspected cases, and some of the older suspected cases, were further confirmed by serology, care should be taken when interpreting these serology results. In most assays, ELISAs were used that were commercially developed to detect anti-PRV antibodies in pig serum, typically detecting antibodies against the viral glycoproteins gB or gE in the context of the DIVA vaccination concept. As far as is known, these ELISAs have not been tested or validated on human sera or other body fluids. Most importantly, PRV is relatively closely related to three highly prevalent human alphaherpesviruses: HSV1, HSV2, and VZV. These viruses have estimated global prevalence rates of 67%, 13%, and 90% for HSV1, HSV2, and VZV, respectively (31, 32). This is of particular concern when detecting antibodies against the viral gB glycoprotein, as gB is one of the most highly conserved glycoproteins in the herpesvirus family (73). Amino acid homology between gB of PRV and its orthologs in HSV1, HSV2, and VZV is 54%, 55% and 56%, respectively. When considering amino acid similarity while allowing conservative amino acid changes, these percentages rise to 71%, 71%, and 70%. The percentages for gE are lower, but still not negligible, with a homology of 29%, 30%, and 28%, respectively, and a similarity of 42%, 45%, and 40%. Importantly, cross-reactivity of antibodies against gB of alphaherpesviruses from different species has been reported before (27), including the recent description of a monoclonal antibody that not only cross-reacts with gB orthologs of alphaherpesviruses from a variety of species but also cross-neutralizes HSV1, HSV2, and PRV (28). In addition, in a study that assessed the presence of anti-PRV antibodies in sera of human encephalitis patients in China with unknown etiology and compared these with anti-PRV antibodies in healthy subjects (period 2021–2017), 3.86% of the healthy cohort tested positive for anti-PRV gB antibodies (74). Although this percentage was lower than that observed in the cohort of encephalitis patients (11.69%), this substantial percentage of positive results in healthy individuals possibly reflects cross-reactivity with anti-HSV/VZV gB antibodies rather than true PRV infection in this healthy population. In this study, as in virtually all other studies, the threshold to consider a human serum positive for anti-PRV gB antibodies was set at a signal-to-noise ratio (S/N) below 0.6 (74). The authors also pointed out the possibility of false positives when using an S/N threshold that is too high and, therefore, based on their own results and other studies, suggested moving the positivity threshold for anti-PRV gB antibodies in human serum from S/*N* < 0.6 to S/*N* < 0.4 (74). Importantly, in their cohort of 156 out of 1,335 samples from encephalitis patients that had an anti-gB S/*N* < 0.6, only 9 had an S/*N* < 0.4 (74), reducing the PRV anti-gB antibody positivity rate from 11.69% to 0.67% when using a threshold of S/*N* < 0.4. None of the sera from the healthy cohort had an anti-gB S/*N* < 0.4. Clearly, it will be necessary to evaluate and validate the performance of commercial PRV antibody detection ELISA kits for use on human sera and, if necessary, depending on potential cross-reactivity with anti-HSV/VZV antibodies, adapt such ELISA assays before they can be used to draw firm conclusions regarding human PRV infection.

In the absence of PRV antibody ELISA assays that have been validated for use on human sera, when performing serology, more trustworthy results may be obtained when assessing PRV-neutralizing antibodies. This reduces, but does not eliminate (28), the risk of cross-reactivity. So far, only three studies have reported the detection of PRV-neutralizing antibodies in sera of suspected human PRV infection cases: one study in the 1980s (reporting low levels of PRV neutralizing antibodies) (36) and two more recent studies (reporting unspecified levels of PRV neutralizing antibodies) (52, 60). PRV-specific serology diagnostics that are validated for use in humans would represent a very important added value, as this would also allow more general testing for the presence of PRV-specific antibodies in larger human cohorts and enable assessment of whether or not PRV may cause asymptomatic infections in humans.

Given the quite dramatically altered pathology of suspected human cases of PRV infection before and after 2011, and the geographically confined localization of the cases since 2011, this raises the question of whether local evolutionary changes in the PRV genome may have resulted in altered virulence and/or host tropism. Although not entirely conclusive, there are indications pointing in this direction. The timing of the recent suspected cases of human PRV infection coincides with increasing reports of large PRV outbreaks in China since 2011. The PRV strains isolated from these outbreaks all belong to genotype II, showing relatively limited but consistent genetic and possibly antigenic differences with the more classical genotype I strains (72, 75, 76). Some, but not all, of these genotype II strains have been associated with increased virulence in pigs and mice (76–81). However, little is known about the molecular determinants that explain such differences in virulence and pathogenesis.

So far, only one PRV strain (hSD-1/2019) was isolated from a human patient (60). This isolate, as well as other variant PRV strains from China, appears to replicate better *in vitro* in human cell lines and displays increased intercellular/syncytial spread in these cells compared to classical genotype I and genotype II PRV strains (82). Very recently, the human-isolated hSD-1/2019 strain was reported to trigger enhanced production of so-called tunneling nanotubes (TNTs) in SK-N-SH human neuroblastoma cells compared to other PRV strains (83). TNTs are actin-based, long cell projections that interconnect distant cells (84). PRV had been reported before to trigger TNT formation via the viral US3 protein kinase (85–88), and PRV-induced TNT formation is associated with enhanced intercellular virus spread *in vitro* (85, 87). Hence, it will be interesting to pursue whether enhanced TNT formation by human-isolated PRV strain(s) increases the virus’ ability to infect and spread in human neurons. More broadly, it will be important to compare different genomic and functional aspects of this human PRV isolate, and possible future human PRV isolates, with classical PRV strains in relevant *in vitro* and *ex vivo* human models, to assess if and how this virus displays an increased ability to infect humans.

In addition, the close association of genotype II PRV strains, particularly strains belonging to the so-called clade 2.2, with severe human encephalitis cases appears to warrant an international surveillance to detect whether similar strains may circulate in other PRV-affected regions in the world and whether or not this is associated with human encephalitis cases. Conversely, especially in regions that are or have been affected by (genotype II) PRV outbreaks, it would be valuable to retrospectively assess encephalitis cases of unknown etiology for the presence of PRV sequence reads in the CSF and/or PRV-specific (neutralizing) antibodies in serum, using still-to-be-developed human-validated PRV serology tests.

While not much is known about potential genetic changes in the PRV genome that may or may not affect its ability to infect and/or cause disease in humans, even less is known about potential host factors that may affect susceptibility to PRV. Indeed, except for the observation that virtually all suspected recent human cases of PRV infection involved (occupational) contact with raw PRV-contaminated pig meat, and that most cases had obvious skin damage at hands or fingers that may have facilitated infection, there is currently no information available as to whether there may be specific host factors that predispose particular individuals for an increased risk of severe PRV-induced disease. One important gap in our current understanding is whether or not the affected patients were infected with HSV1, HSV2, and/or VZV. On the one hand, such infections could increase the risk of misdiagnosis of human alphaherpesvirus-induced encephalitis for putative PRV-caused disease. On the other hand, in the absence of such human alphaherpesvirus infection, it could be worthwhile to further assess whether the absence of putative cross-protective immunity provided by human alphaherpesvirus infection, including cross-reacting antibodies, could contribute to PRV susceptibility.

In addition, for human alphaherpesviruses HSV1 and VZV, the development of viral encephalitis has been linked to specific genetic deficiencies that affect proper functioning of the antiviral innate response, particularly the type I/III interferon and NK cell response (89, 90). The described suspected human cases of PRV encephalitis mostly do not mention particular (immune-related) predisposing health issues or diseases. Nonetheless, it would be interesting to perform *in vitro* functional assays with patients’ samples to compare whether the innate response of these patients against PRV, in particular the innate interferon and NK cell response, is impaired compared to that observed in healthy individuals. More generally, additional research is needed to shed light on the currently ill-understood molecular mechanisms underlying the high resistance of humans and higher primates against PRV infection and/or severe disease, at least in the context of genotype I infection, and the suspected rare breach of such resistance by genotype II strains in specific patients.

It has been reported that genotype I-based vaccines, including the widely used attenuated Bartha vaccine strain, may not always elicit sufficient protection in pigs against challenge with some of the recently circulating genotype II strains (91, 92). Nonetheless, it is noteworthy to point out that several reports have demonstrated adequate protection in pigs elicited by classical PRV vaccine strains like Bartha and Begonia against at least some of these newly emerging strains (93–97). However, it is likely that vaccine strains that are based on the genotype II genome, which are therefore antigenically more closely related to the strains currently circulating in China, may trigger improved immune responses against genotype II strains (98). Different genotype II-based attenuated and inactivated PRV vaccine strains, as well as subunit/mRNA vaccines targeting conserved PRV epitopes and proteins, are being developed and/or have been licensed recently and have shown promising efficacy in PRV genotype II challenge assays in pigs (99–112). Although in several cases the developed vaccines were reported to outperform classical PRV vaccines like Bartha, it has to be pointed out that in the vast majority of these reports, challenge assays were carried out using the homologous genotype II parental strain from which the vaccine was derived. It would be important to also assess the efficacy of some of these promising vaccine candidates in heterologous settings using (antigenically) different PRV strains as challenge strains. In any case, several of the vaccine candidates that are in development and/or recently licensed vaccines may play an important role in the eradication of PRV from genotype II-affected regions.

Irrespective of the PRV vaccines used for this purpose, it appears critical, not only for the pig industry but also from a zoonotic point of view, that regions affected by PRV outbreaks, especially genotype II strains, establish efficient vaccination and eradication programs. In line with this, recent reports have made urgent calls to eradicate PRV from domestic pigs in China (99, 113). Past experiences, particularly in parts of Europe and the USA, have demonstrated convincingly that PRV eradication from the domestic pig population in countries and regions with high pig density and suffering from large PRV outbreaks is realistic and feasible (6, 114). Key aspects of successful eradication include mandatory and appropriately performed vaccination of domestic pigs and the establishment of a fine-grained and careful diagnostic program based on the invaluable DIVA concept to rapidly and efficiently identify infected animals, thereby curbing local outbreaks early and preventing persistent virus circulation and recurrent outbreaks (6, 114). Although eradication programs are particularly challenging to impose in areas with many small to intermediate farms, mandatory and well-controlled vaccination is an essential and crucial step to eradicate PRV from domestic pigs.

In addition to increasing vaccination efforts, it appears important to raise awareness and improve protection against putative PRV infection in individuals at risk. In particular, individuals who have occupations involving frequent contact with raw pork meat in regions experiencing outbreaks of genotype II PRV should have sufficient protective gear, including protective gloves and glasses, to avoid contact with PRV via skin breaches or via the eyes. Furthermore, given the generally lower efficacy of antivirals against PRV compared to HSV (44, 48, 49), the development of antivirals that show high efficacy against PRV and that preferentially display an ability to efficiently cross the blood-brain barrier in humans should be promoted.

In conclusion, despite the concerning surge in cases of suspected human infection with PRV in China over the past years and the severe disease symptoms and poor outcome associated with these suspected cases, it is important to state that the risk of human PRV infection remains very low and that human-to-human transmission has never been reported. Taking all evidence into account, it is advisable to increase awareness and improve protection (mainly hand and eye protection) against possible infection, particularly for people who are occupationally involved in processing raw pig meat in regions with high prevalence of (clade 2.2) genotype II PRV. In addition, it is advisable to initiate stringent programs to eradicate PRV from domestic pig populations, especially in regions/countries experiencing genotype II PRV outbreaks. Based on previous success stories, successful eradication will rely heavily on mandatory and stringent mass vaccination using existing and/or antigenically optimized DIVA vaccines and adequate diagnostic surveillance to rapidly detect and curb outbreaks. Further, in the context of a better diagnosis, treatment, and understanding of potential human infection with PRV, it is important to increase research on several aspects, including the development of PRV diagnostics that are optimized and validated for use on human sera and other body fluids; the development of antivirals that display high efficacy against PRV and that preferentially cross the blood-brain barrier; international surveillance for PRV strains that are particularly linked to human infection; the identification of putative genomic changes in PRV that affect virulence and host tropism; and the identification of putative human host factors that affect susceptibility/resistance to PRV.
